# The antibiotic peptaibol alamethicin from *Trichoderma* permeabilises *Arabidopsis* root apical meristem and epidermis but is antagonised by cellulase-induced resistance to alamethicin

**DOI:** 10.1186/s12870-018-1370-x

**Published:** 2018-08-10

**Authors:** Bradley R. Dotson, Dia Soltan, John Schmidt, Mariam Areskoug, Kenny Rabe, Corné Swart, Susanne Widell, Allan G. Rasmusson

**Affiliations:** 1 Department of Biology, Lund University, Sölvegatan 35B, 223 62 Lund, Sweden; 2 Present Address: Botany Department, Faculty of Science, Sohag University, Sohag, 82524 Egypt; 3 Present Address: MariboHilleshög AB, Säbyholmsvägen 24, 261 91 Landskrona, Sweden; 4 Present Address: Institute of Natural Materials Technology, Technische Universität Dresden, Bergstraße 120, 01069 Dresden, Germany; 5 Present Address: Max Planck Institute of Molecular Plant Physiology, Am Mühlenberg 1, 14476 Potsdam, Germany

**Keywords:** Alamethicin, Arabidopsis, Biotic interaction, Bright yellow 2 cells, Cellulase, Peptaibol, Trichoderma

## Abstract

**Background:**

*Trichoderma* fungi live in the soil rhizosphere and are beneficial for plant growth and pathogen resistance. Several species and strains are currently used worldwide in co-cultivation with crops as a biocontrol alternative to chemical pesticides even though little is known about the exact mechanisms of the beneficial interaction. We earlier found alamethicin, a peptide antibiotic secreted by *Trichoderma*, to efficiently permeabilise cultured tobacco cells. However, pre-treatment with *Trichoderma* cellulase made the cells resistant to subsequent alamethicin, suggesting a potential mechanism for plant tolerance to *Trichoderma*, needed for mutualistic symbiosis.

**Results:**

We here investigated intact sterile-grown *Arabidopsis thaliana* seedlings germinated in water or growth medium. These could be permeabilised by alamethicin but not if pretreated with cellulase. By following the fluorescence from the membrane-impermeable DNA-binding probe propidium iodide, we found alamethicin to mainly permeabilise root tips, especially the apical meristem and epidermis cells, but not the root cap and basal meristem cells nor cortex cells. Alamethicin permeabilisation and cellulase-induced resistance were confirmed by developing a quantitative in situ assay based on NADP-isocitrate dehydrogenase accessibility. The combined assays also showed that hyperosmotic treatment after the cellulase pretreatment abolished the induced cellulase resistance.

**Conclusion:**

We here conclude the presence of cell-specific alamethicin permeabilisation, and cellulase-induced resistance to it, in root tip apical meristem and epidermis of the model organism *A. thaliana*. We suggest that contact between the plasma membrane and the cell wall is needed for the resistance to remain. Our results indicate a potential mode for the plant to avoid negative effects of alamethicin on plant growth and localises the point of potential damage and response. The results also open up for identification of plant genetic components essential for beneficial effects from *Trichoderma* on plants.

**Electronic supplementary material:**

The online version of this article (10.1186/s12870-018-1370-x) contains supplementary material, which is available to authorized users.

## Background

Many agricultural crops are threatened by pathogenic microorganisms against which there is no efficient chemical agent, either because the pathogens have developed resistance or environmental concerns [1]. Biocontrol solutions have therefore been sought after, and especially several species of *Trichoderma* have been widely used. Many species of *Trichoderma* live in the rhizosphere of a vast number of plant species, and often together, e.g. five different species were found in the same rhizosphere of tomato [2]. *Trichoderma* are known to have several beneficial effects on the plant. These include direct antagonistic effects on the pathogens, stimulated pathogen resistance development [1, 3] as well as direct plant growth promotion [4–7]. Therefore, co-cultivation of crop plants with *Trichoderma* strains is nowadays done frequently worldwide [8–10].

One mode of beneficial influence on plants is that *Trichoderma* secretes hydrolytic enzymes such as chitinase and glucanase that attack and degrade the cell walls of pathogens, [2, 11, 12]. *Trichoderma* also secretes membrane-intercalating peptides called peptaibols that act synergistically to the secreted hydrolytic enzymes and induce cell lysis [11].

Peptaibols are linear, 5–21 amino acids long, non-ribosomally synthesised peptides that are rich in alpha-amino isobutyric acid, and that insert into energised membranes when approaching from the net-positive side. One of the peptaibols secreted by *Trichoderma* is the 20-residue alamethicin, which self-associates into narrow voltage-dependent channels [12–15]. Alamethicin has been intensively used as a model molecule to study membrane channel behaviour in defined lipid environments [13] but also with regard to its antibiotic effect on different pathogenic microorganisms [16]. Natural and synthesised peptides are nowadays screened for their antimicrobial specificity regarding pathogenic microorganisms [17–20]. The peptide – membrane interactions are most likely dependent on membrane properties such as charge and lipid composition [14, 18, 21, 22]. Therefore, unlike other antibiotics, peptaibols have relatively general modes of action [23] suppressing resistance development by the pathogen.

Alamethicin has multiple effects on plant cells, depending on concentration. At concentrations below 5 μg ml^− 1^ jasmonate and salicylate elicitation occurs in Lima bean [24] and *Arabidopsis thaliana* root growth is inhibited [25]. Concentrations of 5–20 μg ml^− 1^ induce a non-lethal permeabilisation of the plasma membrane of tobacco cells in 10 min [26], whereas longer incubation or higher concentrations will induce cell death [26, 27]. The exact biological relevance of the peptaibols themselves to the plant is not known, but peptaibols are believed to be involved in the *Trichoderma* parasitism on microorganism [28].

Our earlier finding that alamethicin efficiently permeabilise sterile-grown tobacco cells, and in turn plastids and mitochondria, but not the vacuole, allowed investigation of intracellular enzyme activities [26, 29–31]. This seemed at first conflicting with the fact that *Trichoderma* species often are benevolent to the plant. However, cultured plant cells that were exposed to a commercially available cellulase from *Trichoderma viride* (Onozuka RS from Serva) were found to become resistant to alamethicin permeabilisation [32]. This cellulase preparation is relatively crude [33], but we could conclude that cellulose degradation was needed, since resistance development could be inhibited by cellobiose, the end product of exo/endoglucanase activity, and since boiled enzyme did not induce resistance [32]. Additionally, resistance could not be induced by pectinase (macerozyme), nor by the defence response elicitors xylanase, elf18, flg22 or chitosan. Also an uncoupler and cycloheximide did not inhibit resistance, ruling out the involvement of membrane depolarisation and protein synthesis, respectively [32]. Isolated plasma membranes from resistant cells had a lowered content of phosphatidylserine and a lower sterol to fatty acid ratio [32]. We suggested that this could affect alamethicin channel formation, which is known to depend on the physical properties of the membrane [13, 22]. Thus, these cultured plant cells displayed a clear and unique case that eukaryotic cells can specifically induce resistance to a peptide antibiotic, despite the general nature of alamethicin channel formation [32].

Plant cell cultures may be important for the characterisation of interactions in a heterotrophic system but to investigate the cell specificity and molecular mechanisms behind this Cellulase-Induced Resistance to Alamethicin (CIRA), intact plants with multiple cell types and a defined genome are needed. For successful symbiosis of plants with *Trichoderma* in the rhizosphere it is evident that plant cells need a protection against peptaibols. Based on the extant information, we hypothesises that plant roots contain cells that are sensitive to a peptaibol like alamethicin, that they detect cellulase as a marker for the presence of *Trichoderma*, and they induce a protection against the alamethicin. In this work, we demonstrate that alamethicin permeabilisation and CIRA can be induced in root tip epidermis in the model organism *A. thaliana*. This opens up possibilities to identify key components behind CIRA important in plant-fungus interaction by utilising the molecular tools available for *A. thaliana*.

## Results

### Alamethicin permeabilises *A. thaliana* roots but cellulase induces resistance

**Fig. 1:**
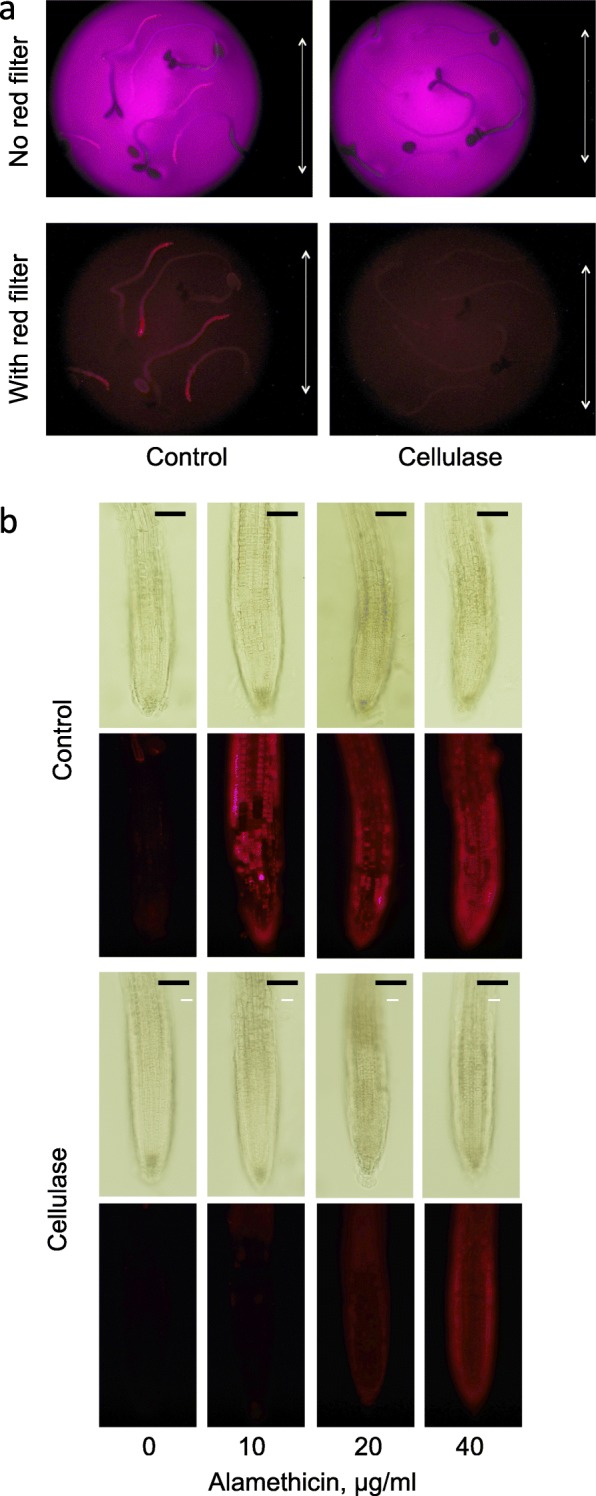
Cellulase pre-treatment of *A. thaliana* seedlings induces resistance to subsequent alamethicin permeabilisation. **a** Seedlings were grown in H_2_O for 4 days. They were then transferred to cellulase in H_2_O or only H_2_O (control), for 4 h, then washed with H_2_O and incubated for 10 min with 20 μg ml^− 1^ alamethicin, and then for 45 s with PrI and quickly washed with H_2_O. Pictures were taken after washing and transfer of seedlings to new medium without alamethicin, using a dissection microscope with or without red filter and with UV excitation. Bar corresponds to 5 mm. The images show one representative replicate out of three. **b** Seedlings were grown in ½ MS for 10 days. They were incubated in cellulase in mannitol or only mannitol (control), for 2 h, and then for 15 min with various concentrations of alamethicin, where PrI was included the last 5 min. Pictures were taken directly using a fluorescence microscope equipped with a G2A filter. Bars correspond to 100 μm. The images show one representative replicate out of three

*A. thaliana* seedlings were found to be permeabilised by alamethicin, as observed using the fluorescent probe propidium iodide (PrI). This hydrophilic dye cannot pass the intact plasma membrane in a normal cell on a few minutes time scale [34], but can after plasma membrane permeabilisation bind to DNA and RNA in the nucleus, ribosomes and organelles. PrI is also commonly used to stain plant cell walls [35], but then using around ten times higher PrI concentrations than what is used for staining nucleic acids. In control seedlings germinated in H_2_O and treated with alamethicin, PrI fluorescence was seen in the young parts of the root but not in other parts of the seedling (Fig. 1a). In contrast, seedlings exposed to a limited, 4 h cellulase treatment showed no such fluorescence (Fig. 1a). Seedlings germinated in ½-concentrated Murashige and Skoog (MS) medium were also exposed to increasing concentrations of alamethicin (Fig. 1b). No fluorescence was observed in the absence of alamethicin, neither with control seedlings nor with cellulase-treated ones (Fig. 1b). With control seedlings alamethicin addition (10–40 μg ml^− 1^) resulted in PrI fluorescence that was most intense in the root tip (Fig. 1b), but fluorescence was only found at the highest alamethicin concentration if the seedlings had been pretreated with cellulase (Fig. 1b). Cellulase pre-treatment also prevented alamethicin permeabilisation further back in the young root, i.e., in the early root hair zone. However, the fluorescence in this zone of permeabilised control seedlings was weaker (Fig. 1a; Additional file 1: Figure S1), probably due to that the cells here are more vacuolated. No PrI fluorescence was observed in the wax-covered tissues of the hypocotyl or cotyledons. This was verified using the YO-PRO probe, which has similar properties as PrI, yet fluoresces green and is not disturbed by chlorophyll (Additional file 2: Figure S2). Taken together, our results show that there are cells in seedling root tips that are permeabilised by alamethicin, independent of germination medium used. This is similar to cultured tobacco cells, which have been shown permeabilised using PrI as a probe, or using enzymatic or polarographic assays [31, 32]. Furthermore, the permeabilisation of the seedling cells could be inhibited by pre-treatment with cellulase, as earlier found with cultured cells [32].

### Alamethicin permeabilisation is most prominent in the meristem and the early extending epidermal cells of *A. thaliana* roots

**Fig. 2:**
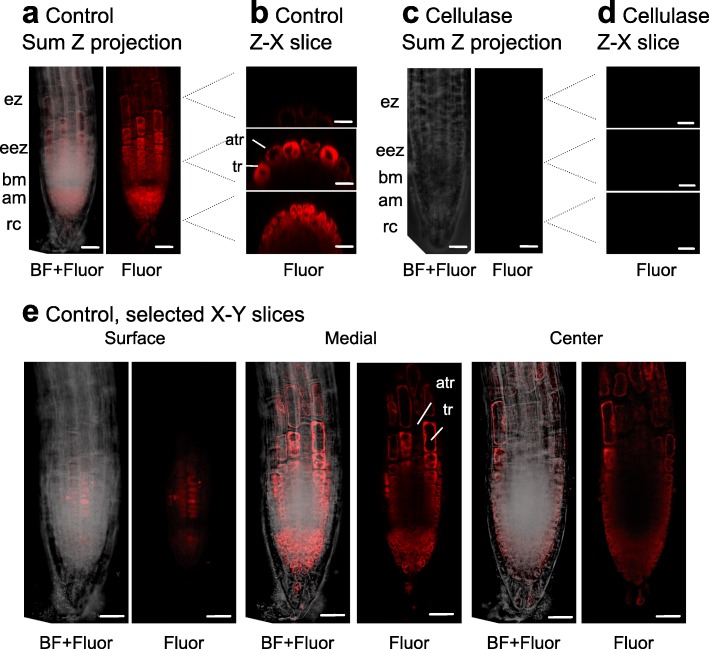
Alamethicin mainly permeabilises epidermal cells of *A. thaliana* seedlings. Seedlings were grown for 5 days in ½ MS. Confocal laser scanning microscopy images were collected as slices through the root in different 2-D planes. **a**, **b**, **e** Control plants, **c**, **d** Cellulase-treated plants. After treatment with cellulase in mannitol or in mannitol only (control) for 3 h, they were incubated for 15 min with 20 μg ml^− 1^ alamethicin in mannitol, where PrI was included the last 5 min. PrI can be seen to stain nuclei and the cytoplasm, whereas cell walls are not stained. Alam, alamethicin; Fluor, fluorescence; BF, bright field; Sum Z projection are the fused Z slices and reflects a compressed 3-D image; Z-X slices are selected images along the y-axis, where the levels analysed are indicated by dotted lines; X-Y slices are selected images along the Z-axis at different depths in the root; rc, root cap; am, apical meristem zone; bm, basal meristem zone; eez, early extension zone; ez, extension zone; atr, atrichoblast; tr, trichoblast. One of three experiments with similar results is shown. Bar corresponds to 25 μm

We next wanted to document whether there were differences in the degree of permeabilisation between the cell types of the root tip. To do this, roots were subjected to confocal laser scanning microscopy (Fig. 2). In control (non-cellulase treated) seedlings, alamethicin-dependent PrI fluorescence was seen in the nuclei and cytoplasm, but not cell wall, in the root apical meristem and extension zone, yet cell staining decreased with increasing vacuole size further up in the extension zone (Fig. 2a and b). No staining was seen in the root cap (Fig. 2a). The cross sections showed that most cells in the meristematic zone showed fluorescence (Fig. 2b lower), whereas in the early extension zone only the epidermal cells did (Fig. 2b middle). It seemed that trichoblasts were more stained than atrichoblasts both as seen in the cross sections (Fig. 2b middle), and the longitudinal sections (Fig. 2e). Interestingly, less permeabilisation was repeatedly seen in the basal meristem zone located between the apical meristematic zone and the early extension zone (Fig. 2a, b and e). Again, no fluorescence was seen with cellulase-treated material (Fig. 2c and d).

### Alamethicin permeabilisation can be determined enzymatically in tobacco cells and *A. thaliana* seedlings using NADP-dependent isocitrate dehydrogenase

With tobacco cells, alamethicin-permeabilisation and resistance have been successfully monitored as PrI fluorescence, as well as using biochemical markers [32]. We therefore searched for an enzymatic marker suitable for seedlings, to get quantitative data that may confirm the fluorescence results. In this respect, NADP-dependent isocitrate dehydrogenase (NADP-ICDH) [36, 37] seemed promising, since the activity is abundant and totally dependent on the presence of Mg^2+^, which easily can be complexed by the addition of EDTA. Furthermore, NADP-ICDH can easily be assayed spectrophotometrically as the reduction of NADP^+^ to NADPH (in the presence of inhibitors for NADPH reoxidation), and is stable during purification.

**Fig. 3:**
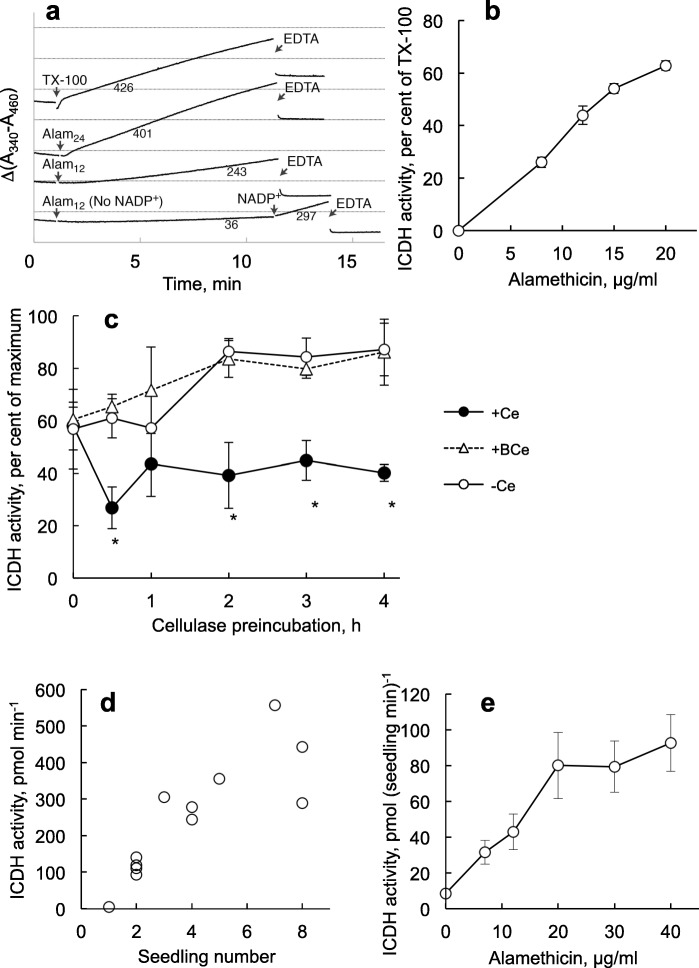
Permeabilisation with alamethicin can be monitored as NADP-dependent ICDH activity. **a** Online spectrophotometric data obtained using *N. tabacum* cell cultures. Numbers below the slopes indicate NADP-ICDH activity rates (nmol min^− 1^ g^− 1^ (fresh weight)). Subscripts indicate alamethicin (Alam) concentration (μg ml^− 1^). Triton X-100 (TX-100) was added to 0.1% (*w*/*v*) and EDTA to 25 mM. The assay shows representative activity patterns among 2–7 separate measurements of each kind. Absorbance difference between horizontal lines, 0.2 A. **b** The effect of alamethicin on the ICDH activity in *N. tabacum* cell cultures (20 mg ml^− 1^). The reaction was stopped after 10 min using excess EDTA and measured using a plate reader. Maximum is activity in the presence of 0.1% (w/v) Triton X-100. Bars show SE for *n* = 3. **c** ICDH-activity after alamethicin permeabilisation in cells (20 mg ml^− 1^) preincubated with 0.2% (w/v) cellulase (+Ce) or boiled cellulase (+BCe). Activity was measured as in (**b**) using 12 μg ml^− 1^ alamethicin. Bars show SE for *n* = 3. **d** ICDH activity using 40 μg ml^− 1^ alamethicin, in different numbers of *A. thaliana* seedlings that had been grown for 5 days in H_2_O. Reaction was stopped after 1 h with excess EDTA. **e** ICDH activities in seedlings, as in (**d**), with different concentrations of alamethicin. Bars show SE for *n* = 4

Upon addition of 24 μg ml^− 1^ alamethicin to control tobacco cells, NADP-ICDH activity could be detected after a short lag-phase of ca. 1 min, whereas a 12 μg ml^− 1^ alamethicin gave a ca. 5 min lag phase before a linear rate was observed. The rates were comparable to the rate evoked by addition of 0.1% Triton X-100 and activity was efficiently stopped by addition of excess EDTA (Fig. 3a). (The drop in absorbance after EDTA addition probably reflects a change in scattering induced when Mg^2+^ was complexed by EDTA since it is similar for all curves.) The linearity of the reaction and the flat curve after adding EDTA shows that NADPH was not degraded in the assay. No alamethicin-induced absorbance change was seen in the absence of NADP^+^ (Fig. 3a). Having tested this online-assay, we scaled up the procedure to be used in microtiter plates where the activity was stopped with EDTA after 10 min. The activity reached after permeabilisation with alamethicin was 70–90% of that obtained with 0.1% Triton X-100 (Fig. 3b) which agrees with on-line measurements (Fig. 3a) and earlier studies on cytosolic enzymes in tobacco cells [26]. Half maximal NADP-ICDH activity was found with 10–12 μg ml^− 1^ alamethicin at a cell concentration of 20 mg cells ml^− 1^ (Fig. 3b).

Incubation of cells with 0.2% cellulase resulted in alamethicin resistance that was complete after 3 h (Fig. 3c). Thus, it seemed that NADP-ICDH activity seemed to be a quick assay that reliably could be scaled up for measuring permeabilisation and cellulase-induced resistance to alamethicin for tobacco cells. We next tested whether this was true also for *A. thaliana* seedlings.

NADP-ICDH showed a clearly detectable activity in *A. thaliana* seedlings after that these had been treated with alamethicin. The activity was stable over extended assay times up to at least 60 min, and could therefore be detected in few seedlings, increasing linearly with number of seedlings in the assay (up to at least 6; Fig. 3d). The pattern of the alamethicin dependency was similar to that obtained with cells (compare Fig. 3b and e).

**Fig. 4:**
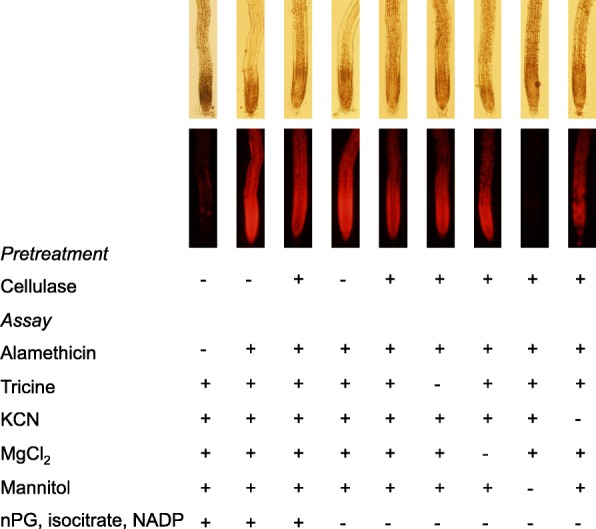
Cellulase-induced resistance in *A. thaliana* seedlings depends on the osmoticum during permeabilisation. Seedlings grown for 5–6 days in H_2_O and pretreated ±cellulase in H_2_O were transferred to different media. Fluorescence images were collected using PrI to detect permeabilisation by alamethicin (20 min, the last five with PrI probe present). Three hours Cellulase, 1%; Alamethicin, 20 μg ml^− 1^; Mannitol, 0.35 M; Tricine, 50 mM, pH 8; MgCl_2,_ 3.0 mM; KCN 1.0 mM; nPG, n-propyl gallate 100 μM, isocitrate, 2 mM, NADP^+^ 1 mM. The images show one representative replicate out of three

**Fig. 5:**
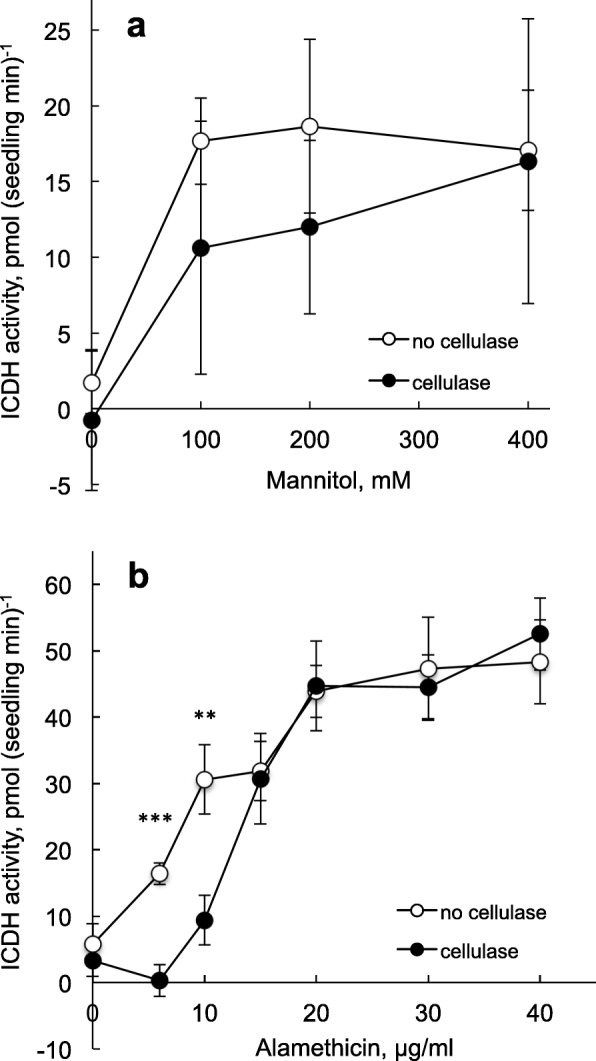
CIRA detected in *A. thaliana* seedlings by NADP-ICDH assays. Seedlings were grown for 5 days as in Fig. 4. Data on NADP-ICDH activity was collected using a plate reader. **a** The effect of mannitol assayed with 13 μg ml^− 1^ alamethicin. Mean values from four different experiments where *n* was 5–10 for each treatment within each experiment. Bars show SE. **b** The effect of alamethicin assayed in the presence of 100 mM mannitol. Average values ±SE for *n* = 6–11 seedling pools are shown. Final concentrations besides mannitol and alamethicin were as for Fig. 4. Significant differences ± cellulase are denoted with asterisks: ***, *p* < 0.001; **, *p* < 0.01

Initially, we could not detect CIRA with seedlings when analysing NADP-ICDH activity. This was confusing since it disagreed with the fluorescence data for roots (Figs. 1 and 2) and with the observations that tobacco cells showed alamethicin resistance using both PrI fluorescence [32] and NADP-ICDH activity as markers for permeabilisation (Fig. 3c). However, the medium used in the NADP-ICDH assay is more complex than the one used for fluorescence assays, and it could not be excluded that the water-grown seedlings could react more strongly to this than the cells that were cultured in MS medium + sucrose. As a first approach we therefore set out to test the components in the NADP-ICDH assay medium in more detail, using PrI fluorescence as permeabilisation marker. Indeed, no cellulase-induced resistance was obtained in the complete medium, but appeared when mannitol was deleted from the medium (Fig. 4). We then returned to the NADP-ICDH activity assay, and found that pre-treatment with cellulase resulted in alamethicin resistance provided that the mannitol concentration in the subsequent assay was lowered (Fig. 5a). Using 100 mM mannitol, alamethicin resistance could be detected in seedlings, and we determined that a two-fold higher alamethicin concentration was needed for permeabilisation after cellulase treatment (Fig. 5b). Taken together, our results show that cellulase pre-treatment can induce cell-type specific CIRA in sterile-grown *A. thaliana* seedlings. We show this with two separate methods (fluorescence and enzyme activity) and with two growth conditions (H_2_O and ½ MS). The presence of CIRA in *A. thaliana* opens up possibilities to investigate the genes that take part in the mechanisms of CIRA by molecular tools, furthering understanding of the interaction between the commonly used biocontrol fungus *Trichoderma* and plants.

## Discussion

*Trichoderma* species act in three interconnected ways to increase the wellbeing of plants. They cause direct growth promotion [4–6], they elicit induced systemic resistance development in the plant [1, 3, 9, 38] and they also secrete e.g., enzymes [8] and peptides [16] that directly act on the wall and the plasma membrane, respectively, of surrounding pathogens to lyse their cells. However, it has been reported that the *Trichoderma* effect on plant growth can vary with plant and fungal genotype, and even lead to growth inhibition in some cases [39, 40]. This suggests that *Trichoderma* can damage plants, as also is indicated by alamethicin permeabilisation of plant cells (Fig. 1) [26], and inhibition of plant root growth [25] by concentrations at or below those inhibiting microorganisms [41–43]. The peptaibols released by various *Trichoderma* spp. have a relatively general mode of action on plants, as indicated by the *A. thaliana* mutant *tkr1*, which provides resistance to growth inhibition by the peptaibols trichokonin VI and alamethicin [25]. Peptaibol lysis of plant cells could thus counteract the benevolent property of *Trichoderma* fungi. However, pre-treatment of plant cells with *Trichoderma* cellulase induces the CIRA resistance to the subsequent action of alamethicin (Fig. 1) [32]. Presence of cellobiose, the end product of exo/endoglucanase activity, prevented CIRA development in tobacco cells, showing that active glucanases were essential components in the Onozuka RS enzyme preparation used [32]. Several other biotic factors were also tested and found to be inefficient, such as pectinase as well as elicitors such as xylanase, elf18, flg22 and chitosan [32]. During the CIRA induction the plasma membrane lipid composition changed, indicating that these membrane properties were linked to the inhibition of alamethicin pore formation [32]. A possible mechanism would be that a component derived from the action of *Trichoderma* cellulase on the plant cell walls, somehow led to membrane lipid changes so that the plant cells tolerate the alamethicin. This would fit with a benevolent role of *Trichoderma* for plant growth, at least as long as similar defence responses are not initiated in the pathogen. It is important to note that the cellulase treatment used only cause a minor degradation of the plant cell wall, which is hardly visible by microscopy (Figs. 1 and 2) [32] . The concentration of cellulases in the restricted space between tissue-infiltrating hyphae and intact plant cell walls during *Trichoderma*-root interactions is presently unknown. However, *Trichoderma* genes for cell wall-degrading enzymes, including glycosyl hydrolases, are induced during colonization of plant roots, and a setup of *Trichoderma virens* secreted glycosyl hydrolase gene products have been detected in the apoplastic space of maize roots [44, 45]. It is thus plausible that the CIRA process may be one of the communication paths needed for successful plant-fungal symbiosis.

In the present investigation we used seedlings of *A. thaliana* for our studies on permeabilisation and CIRA, to get closer to an intact and natural system than is possible in cultured cells. This approach also made it possible to identify the effect of the permeabilisation process on defined cell types and tissues, something that cannot be done using cell cultures.

Alamethicin permeabilised root cells and cellulase induced resistance (Fig. 1) at similar concentrations and conditions as was previously needed for tobacco cells [32]. Interestingly, all tissues and cells could not be equally permeabilised by alamethicin. We found that especially the root tips were permeabilised and this was observed in various conditions of growth and assays (Fig. 1), suggesting that young and actively expanding cells were more sensitive to alamethicin than differentiated cells. A more detailed confocal microscopy analysis of the root tip showed that meristematic and extending epidermal cells were permeabilised. In contrast, the extending cortex cells, as well as the cells of the root cap and the basal meristem were not permeabilised. The cells of the columella (in the root cap) and the basal meristem have stopped dividing and begun vacuolisation and are therefore quite different compared to the cells of the apical meristem (for a review on spatial regulation of root growth, see [46]). This probably also regards the trafficking of lipids and proteins to the plasma membrane. Using a fluorescent probe, di-4-ANEPPDHQ on *A. thaliana* roots, membrane lipid order of the basal meristem (root transition zone) was found to be higher than in other parts of the root [12]. Also, the lipid order was higher in the cortex as compared to the epidermis [12]. Thus, there is a difference in membrane lipid composition along the root axis that may explain why different parts are differently permeabilised by alamethicin. This would be in line with that CIRA coincided with changes in plasma membrane lipid composition of tobacco cells [32]. Another possible explanation may be found in the cell wall/extracellular environment and how easily the hydrophobic alamethicin as well as the hydrophilic PrI can be transported through it. The cells of the basal meristem (transition zone) are characterized by e.g., specific pectic substances [47] and the root cap secretes hydrophilic mucilage, which may form a diffusion barrier to alamethicin.

The epidermal cells were permeabilised whereas only little PrI fluorescence was observed in deeper tissues at least under the conditions used here (20 μg ml^− 1^ alamethicin for 15 min). PrI, at least at a 10-fold higher concentration used for staining cell walls, can diffuse through the apoplast until the endodermal barrier [48]. This indicates that alamethicin is not efficiently transported apoplastically, nor via plasmodesmata, but then alamethicin induces callose plugging of the latter [31]. Also, alamethicin traversing the apical plasma membrane from the apoplast into the cytosol should not be able to permeabilise the inner, basal plasma membrane of the epidermal cells when approaching from the cytosolic, net negative side of the membrane [26]. Further there seemed to be less staining in atrichoblasts compared to trichoblasts (Fig. 2). The latter difference was non-quantitative, and should probably be interpreted with some care since the atrichoblasts are more vacuolated than trichoblasts and have relatively less cytoplasm (with e.g. organellar nucleic acids) and would therefore display a lower maximum fluorescence level per unit surface.

With tobacco cell cultures, several enzymes were investigated in parallel to PrI fluorescence with regard to CIRA, and the results were in agreement [32]. The advantage of the biochemical assays is that they give more quantitative data and can therefore be used also to find differential changes in permeabilisation and CIRA, whereas PrI staining appears mainly qualitative. Our question was then whether a similar biochemical approach could be used with seedlings. We chose to follow the cytosolic enzyme NADP-ICDH and to do so in microtiter plates for higher throughput. The NADP-ICDH is a house-keeping enzyme with mainly a cytosolic location, and it shows high and constant activity in different parts of the plant and is robust upon purification [36, 37]. The activity of NADP-ICDH in cytosols is much higher than the transport flux via the alamethicin pore that is needed for PrI labelling of intracellular nucleic acids. Therefore, the transport event will be the limiting step for the measured rate of NADPH formation, as was also confirmed by the sigmoidal concentration curves, which are typical for alamethicin (Fig. 5). CIRA induction could be found also using the NADP-ICDH-based assay, despite the much longer incubation times that were needed for detecting NADP-ICDH in few seedlings, as compared to cultured cells. For CIRA to be enzymatically detected with seedlings, the concentration of the osmoticum (mannitol) during assay had to be lowered as compared to when measuring on cells. The difference probably reflects their respective prior growth conditions, i.e., the cells were cultured in a 1xMS medium with 88 mM sucrose, and the seedlings were cultured in H_2_O. It appears that plasmolysis during the NADP-ICDH assay counteracts prior cellulase-induced resistance, suggesting that components linking the plasma membrane to the cell wall may be important for CIRA to be efficient. One possibility would be arabinogalactan proteins that are anchors between the cell wall and the plasma membrane, where e.g., a GFP-AtAGP18 fusion protein could be found in the plasma membrane as well as in Hechtian strands after plasmolysis in tobacco Bright Yellow 2 cells [49].

## Conclusions

In this investigation, we show that alamethicin permeabilisation and CIRA occurs in *A. thaliana* seedling root tips. Permeabilisation takes place specifically in the apical meristem and extension zone epidermis, localising the target cells for alamethicin damage to roots. Plasmolysis inhibition of CIRA indicates that CIRA is dependent on plasma membrane-cell wall contact. The presence of the CIRA process in the model plant *A. thaliana* also opens up possibilities to identify mechanistic components behind alamethicin resistance, and how CIRA may be connected to the usually beneficial role of *Trichoderma* in crop plants.

## Methods

### Cell cultures

*Nicotiana tabacum*, L., Bright Yellow 2 cells were grown in MS basal medium [50] supplemented with 88 mM sucrose, 0.9 μM 2,4-dichlorophenoxy-acetic acid, 3 *μ*M thiamine, 0.5 mM *myo*-inositol and 2 mM KH_2_PO_4_ as described [26]. The initial pH of the medium was 5.0. The cell suspension was subcultured every seventh day and the cells were harvested for experiments during their exponential growth phase (350–450 mg cells (fresh weight) ml^− 1^).

### Plant cultures

Seeds of *A. thaliana* Col-0 (Lehle Seeds, Round Rock, TX, USA) were surface-sterilised and transferred to micro centrifuge tubes or 3.5 cm petri dishes for cultivation in either ½ MS basal medium (pH 5.65), or in H_2_O. After 2–3 days at 4 °C, the seedlings were transferred to a growth room (16 h light, 24 °C). These seedlings were used for fluorescence microscopy assays after 4–10 days of growth, as specified in the legends.

Alternatively, seeds were sterilised as above, and transferred to 96-well polypropylene plates with lids (Greiner Bio-One, BioNordika, Stockholm, Sweden) with 100 μl sterile H_2_O and ca. 5–10 seeds/well. After 2 days at 4 °C, the seedlings were transferred to the growth room (16 h light, 24 °C). These seedlings were used for NADP-ICDH assays after 5 days of growth.

### Fluorescence microscopy with *A. thaliana* seedlings

After growth, the surrounding medium of the seedlings was replaced with the same volume of H_2_O (for H_2_O-grown seedlings) or 0.35 M mannitol (for ½ MS-grown seedlings), ± 1% (*w*/*v*) cellulase and incubated under gentle agitation for 2–4 h. After this incubation, 0–40 μg ml^− 1^ alamethicin (final concentration) was added, the seedlings were incubated for 10–15 min, where 1.5 μM PrI was included for the last for 1–5 min, after which the seedlings were washed and transferred to microscope slides with PrI but not alamethicin (unless stated otherwise).

Fluorescence microscopy was performed using a G-2A-filter (excitation at 510–560 nm, emission above 590 nm) coupled to a Nikon-Optiphot-2 microscope (Nikon Corporation, Tokyo, Japan). As a reference, a bright field transmission microscopy image was registered. Images were collected with an Olympus DP-70 digital camera (Olympus Optical, Tokyo, Japan). The exposure times were chosen from control roots treated with 20 μg/ml alamethicin and where the nuclei clearly were visible but the background was as dark as possible, to make the staining specific for permeabilised cells. This exposure time was then used for collecting all pictures within the experiment.

Alternatively, fluorescence microscopy was performed using an Olympus CZ-CTV dissection microscope (Olympus Optical, Tokyo, Japan). A UV-trans illuminator (UV Herolab UVT-20 M filter 312 nm, Herolab, Wiesloch, Germany) was used as excitation source and emission was collected using a light red filter (Red23A, Tiffen, Hauppage, NY, USA).

Confocal fluorescent microscopy analysis was performed on 4–5 day old seedlings treated similarly as above. PrI staining was detected with a Zeiss LSM 510 confocal microscope with META detector (ZEISS company, Oberkochen, Germany) equipped with a diode-pumped solid-state 561 nm laser and set to detect 590 nm emission and above. Both bright field and fluorescent images were taken at 1 μM intersections with identical microscope settings. Post image processing of 3D image reconstruction, Z-plot projections, and transverse/lateral optical sections were achieved by ImageJ 1.48v software (NCBI). One composite image containing all fluorescent images was adjusted for intensity to set between background and highest intensity for comparison between all conditions. Bright field images were inverted to outline cells for tissue reference.

### NADP-ICDH assay with tobacco cells

For spectrophotometric online determination of activity, the measurements were done with 20 mg ml^− 1^ of 5-day old tobacco cells, in Reaction medium (final concentrations in assay: 50 mM Tricine/KOH, pH 8, 0.35 M mannitol, 3 mM MgCl_2_, 1 mM EGTA, 1 mM KCN, 100 μM n-propyl gallate, 1 mM NADP^+^, 2 mM D,L-isocitric acid). After stabilisation of the signal, alamethicin, Triton-X-100 and EDTA were added as described in the Fig. legends. A_340_–A_465_ was measured using an Aminco-Olis DW2 Dual Wavelength Spectrophotometer (Olis, Bogart, USA).

For assays in microtiter plates, 5-day old cells were pelleted, washed and resuspended in 0.35 M mannitol. Fifty μl cells (6 mg fresh weight) in 0.35 M mannitol were transferred to wells of a 96 well polystyrene microtiter plate (Greiner Bio-One, BioNordika, Stockholm, Sweden) and the reactions started by the addition of 100 μl 1.5 × Reaction medium (giving a final alamethicin concentration of 0–20 μg ml^− 1^). The reaction was stopped after 10 min by addition of 50 μl 100 mM Na_2_EDTA, pH 8.0 to chelate the Mg^2+^. A_340_ – A_465_ was measured on each well using a 96 well plate reader (Labsystems, Multiskan, Helsinki, Finland). Four to five technical replicates were done for each treatment, and the data presented are the mean of 2–4 biological replicates as described. The enzyme activity was calculated using the NADPH extinction coefficient 6.2 mM^− 1^ cm^− 1^.

Cellulase treatment of cells: Cells were incubated with fresh or boiled (5 min, 96 °C) desalted cellulase (0.2% *w*/*v*) in 0.35 M mannitol. Cells treated with 0.35 M mannitol were used as control in tubes. Samples were withdrawn at different time points after 0–3 h and transferred to polystyrene microtiter plates and mixed with 1.5 × Reaction medium (with 0.25 M mannitol and final concentration of 12 μg ml^− 1^ alamethicin) and assayed for NADP-ICDH activity as above.

### NADP-ICDH assay with *A. thaliana* seedlings

Seedlings grown in 100 μl H_2_O in 96-well polypropylene plates were washed and treated ±1% (*w*/*v*) cellulase, pH 5.0, for 3 h. The cellulase was then exchanged with 50 μl 0–400 mM mannitol, followed by 100 μl 1.5 × Reaction medium (final concentration as for cells, but with 0–400 mM mannitol and 0–40 μg ml^− 1^ alamethicin). The reaction was stopped after 60 min with 50 μl 100 mM Na_2_EDTA. To avoid light scattering from the seedlings, absorbance readings were done after transferring 100–150 μl reaction solution from each well to corresponding wells of a polystyrene plate. Four to eight replicates were done for each treatment, and the data presented are the mean of four separate experiments. A_340_ – A_465_ was measured on each well after transfer of samples to polystyrene plates using a 96 well plate reader (Multiskan GO, Thermo Scientific, Waltham, MA, USA).

### Miscellaneous

*T. viride* cellulase (16,420, ‘Onozuka’ Cellulase RS) was purchased from Serva (Heidelberg, Germany). Alamethicin (A-4665 ‘from *T. viride*’) was from Sigma (St. Louis, MO, USA). The strain used (NRRL 3199) for alamethicin purification has been reidentified as *Trichoderma arundinaceum* [51], but is still named *T. viride* by the producer. PrI-detected permeabilisation of root tips was confirmed using *T. viride* alamethicin from two other suppliers (Additional file 1: Figure S1).

Statistical analysis was performed using Student’s 2-tailed unpaired t-tests in Excel (Microsoft).

## Additional files

Additional file 1: **Figure S1.** Permeabilisation of *A. thaliana* seedlings with alamethicin from different sources. (PDF 848 kb)

Additional file 2: **Figure S2.** Alamethicin permeabilisation of *A. thaliana* seedlings detected with YO-PRO. (PDF 805 kb)

## Abbreviations

CIRA: Cellulase-induced resistance to alamethicin
MS: Murashige and Skoog medium
NADP-ICDH: NADP-dependent isocitrate dehydrogenase
PrI: Propidium iodide
