# Supplementary material for: The antibiotic peptaibol alamethicin from Trichoderma permeabilises Arabidopsis root apical meristem and epidermis but is antagonised by cellulase-induced resistance to alamethicin
Source: BMC Plant Biol. 2018 Aug 10;18:165. doi: 10.1186/s12870-018-1370-x (PMC6086028; doi:10.1186/s12870-018-1370-x)
Supplement: Supplementary file 1 — Figure S1. Permeabilisation of A. thaliana seedlings with alamethicin from different sources. (PDF 848 kb) [file 12870_2018_1370_MOESM1_ESM.pdf]

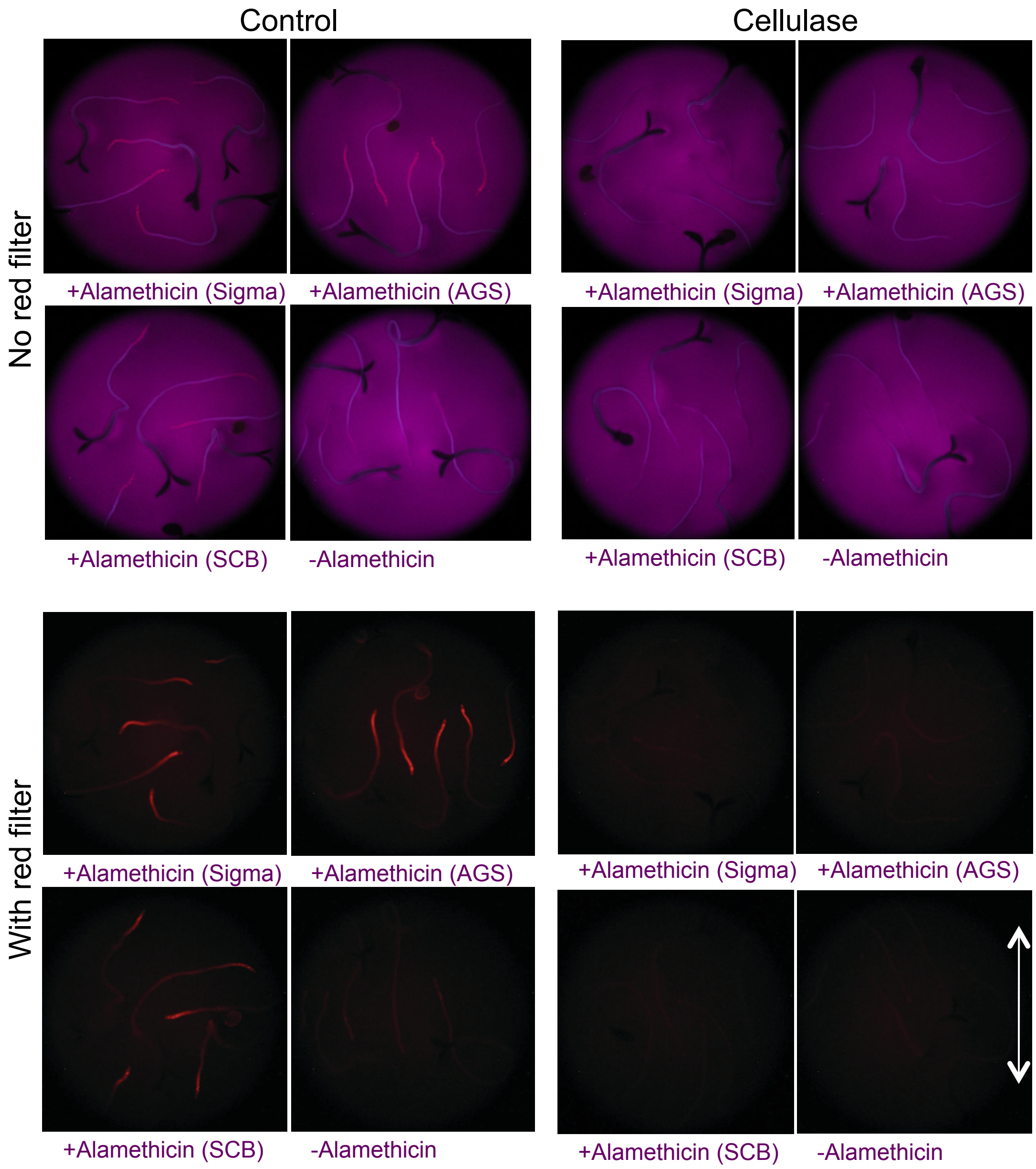

Fig. S1 Permeabilisation of *A. thaliana* seedling with alamethicin from different sources

Seedlings were grown, pre-treated with cellulase, permeabilised, stained, and documented as described for Fig.1, except using H_2O in all steps instead of ½ MS and mannitol solution. Alamethicin from Sigma, A.G. Scientific (AGS) and Santa Cruz Biotechnology (SCB) were used. Bar corresponds to 5 mm. The images show one representative replicate out of two.
