# Supplementary material for: The antibiotic peptaibol alamethicin from Trichoderma permeabilises Arabidopsis root apical meristem and epidermis but is antagonised by cellulase-induced resistance to alamethicin
Source: BMC Plant Biol. 2018 Aug 10;18:165. doi: 10.1186/s12870-018-1370-x (PMC6086028; doi:10.1186/s12870-018-1370-x)
Supplement: Supplementary file 2 — Figure S2. Alamethicin permeabilisation of A. thaliana seedlings detected with YO-PRO. (PDF 805 kb) [file 12870_2018_1370_MOESM2_ESM.pdf]

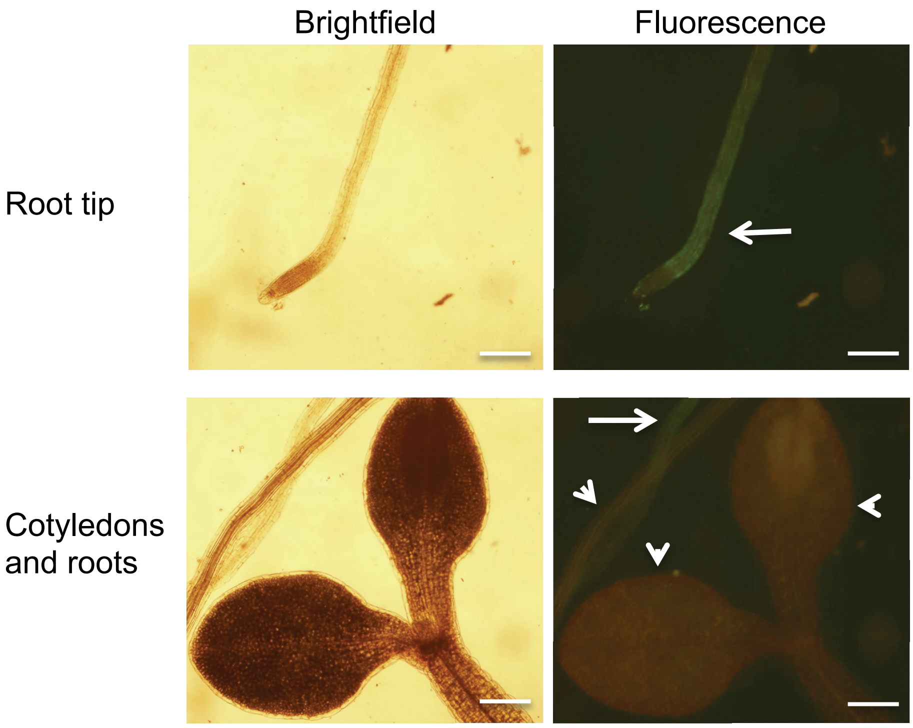

Fig. S2 Alamethicin permeabilisation of *A. thaliana* seedlings detected with YO-PRO

Seedlings were grown in H_2O for 5 days, permeabilised 30 min in 20 µg/ml alamethicin in mannitol medium, and stained by supplementing with 0.1 µM YO-PRO for 45 s. Fluorescence was detected using a B2A filter (excitation 450-490 nm, emmision >520 nm). Green fluorescence is seen in younger roots (arrows) but only a faint red autofluorescence in older roots and cotyledons (arrowheads). The images show one representative replicate out of two. The length of the scale bars is 200 µm.
